# Synthesizing Sex/es Across Scales and Lineages: Centering Plants, Algae, Fungi, Protists, and Modular Metazoans

**DOI:** 10.1093/icb/icag127

**Published:** 2026-07-23

**Authors:** A M Aramati Casper, Karen M Warkentin

**Affiliations:** Biology Department and Graduate Degree Program in Ecology, Colorado State University, Fort Collins, Colorado 80545, USA; Department of Biology, Boston University, 5 Cummington Mall, Boston, MA 02215, USA; Center for Animal Behavior, Smithsonian Tropical Research Institute, Apartado Postal 0843-03092, Panamá, República de Panamá

## Abstract

As humans, we bring biases based on our own vertebrate, mammalian biology into the way we conceptualize the world. This includes how we discuss the range of concepts we reference with the term *sex* and the implicit assumptions that are often embedded in how we discuss life cycles and reproduction. These assumptions situate diplontic life cycles, unitary body plans, and amphigonic reproduction as normative and frame sex (meiosis and syngamy) and reproduction (the creation of new individuals) as inherently intertwined. We draw from the social science theoretical framework of master narrative theory to discuss these implicit assumptions. Master narrative theory typically is used to articulate normative expectations for humans in specific sociocultural contexts. Here we use it to reveal normative expectations about organisms that are common in western modern science. Following this explication, we discuss frameworks and language that biologists working with modular metazoans and plants have developed to discuss complex sexual variation, addressing multilevel modularity and continuous, quantitative variation in investment into or reproductive success via male and female gametes. We consider ways these “specialized” approaches can be useful for broadly comparative work, across organisms with a range of sexual simplicity and complexity, and identify challenges to greater conceptual integration. As a step toward tools for improving conceptual integration across sexual systems and body plans, we introduce a graphical system for mapping anisogamete-production strategy across levels of modularity. We call for further conversations that build on the “Sex Across Origins” symposium at the 2026 Society for Integrative Biology conference, considering key concepts in the biology of sex and reproduction through the prism of different kinds of organisms. This practice can help break apart common oversimplifications and overgeneralizations, reveal the breadth or limits of concepts, and generate insights from the ways that concepts fail for subsets of organisms, suggesting paths forward to better understand and articulate the complexity and variation of sex and reproduction.

## Introduction

There is broad variation in the meanings assigned to the term *sex* throughout the field of biology; this breadth is demonstrated by many other papers in this issue, as well as discussions elsewhere (e.g., Sanz 2017; Fausto-Sterling 2019; Hyde et al. 2019; Maney et al. 2020; Morgenroth and Ryan 2021; Anderson and Falk 2023; Goymann et al. 2023; Massa et al. 2023; McLaughlin et al. 2023; Smiley et al. 2024; Eppley et al. 2026). In this issue, Warkentin et al. (2026), McDonough-Goldstein et al. (2026), and Massa (2026) discuss challenges and incoherences in regards to the many ways *sex* is used and the socio-cultural constructs that have influenced how both *sex* and *gender* are used. From this conceptual breadth, many of the other papers discuss heterogeneity across taxonomic groups, scales of consideration, and conceptual perspectives important in thinking about sex (Billiard et al. 2026; Van Etten and Johnson 2026a; Constable and Liu 2026; dos Santos et al. 2026; Falk 2026; Lerch 2026). The wide variation in biology that is encapsulated across these manuscripts is influenced by many things, including life cycles, modes of reproduction, unitary or modular organization, organismal mobility, and environmental context. And, each of these characteristics, and more, interact across evolutionary, developmental, and ecological contexts.

The many ways the term *sex* is used makes it challenging to even discuss the complexity of sex. Here we use Warkentin et al.’s (2026) conceptual framework that numbers different sex concepts to facilitate clarity in communication and conceptualization (Table 1; see Warkentin et al. (2026) for further descriptions, figures, and examples.) In this framework, sex0 is horizontal gene transfer, sex1 is meiosis and syngamy, sex2 is anisogamy, sex3 is an organism’s anisogamete production strategy, sex4 is the set of traits associated with the anisogamete type/s produced by an individual, sex5 consists of actions that facilitate syngamy, and sex6 consists of the nonreproductive expressions and evolutionary derivatives of syngamy-facilitating actions (i.e., of sex5). The other papers in this issue address many different meanings of *sex*, demonstrating the utility of this conceptual framework. For example, Van Etten and Johnson (2026a) discuss horizontal gene flow (sex0), Constable and Liu (2026) discuss the organization of meiosis and syngamy (sex1) and nonsexual reproductive processes, Falk (2026) describes the interactions between gamete type (sex3) and other associated traits (sex4) in conceptualizing sex as a polymorphism, and Lerch (2026) considers how classic theory about mate choice (sex5) is highly informative in understanding the evolution of same-sex sexual behavior (sex6). Therefore, the range of papers in this volume demonstrates some of the different ways sex concepts manifest across perspectives and contexts.

**Table 1:** Seven sex concepts with descriptions, based on Warkentin et al. (2026).

Sex #	Concept	Description
Sex0	Horizontal gene transfer	DNA transfer between closely or distantly related organisms by mechanisms other than descent. HGT occurs among and between bacteria, archaea, animals, plants, fungi, and more; more frequent in prokaryotes than eukaryotes.
Sex1	Meiosis and syngamy	Eukaryotic cytogenetic processes involving DNA repair and mixing, epigenetic reset, and cyclic ploidy change. Sexual nuclear, and usually cell, division that halves ploidy and mixes parental genomes; sexual cell contact, and usually fusion, that doubles ploidy by uniting two parental genomes in a single cell and nucleus.
Sex2	Anisogamy	Binary gamete size difference linked with compatibility, so that syngamy only occurs between macrogamete–microgamete pairs. Evolved independently in many lineages, including in the common ancestor of plants and that of animals. Prevalent throughout those two clades; sometimes lost in others.
Sex3	Anisogamete-production strategy	Female (produces only macrogametes), male (produces only microgametes), or hermaphrodite (produces both macrogametes and microgametes). Some species include females and males, some include only hermaphrodites, and others include mixtures of hermaphrodites with females and/or males.
Sex4	Traits associated with anisogamete type/s produced	Lineage-specific set of genetic and phenotypic traits that are associated, although generally not perfectly, with gamete type/s produced. Can include, for instance, genes, chromosomes, gonad or gametangia type, hormonal profile, many aspects of anatomy, coloration, size, behavior, etc.
Sex5	Syngamy-facilitating actions and interactions	Inter/actions of gamete producers that bring gametes together to enable syngamy. Includes animal mating as well as many other kinds of inter/actions that bring gametes together in space and time, such as synchronized broadcast spawning, spermcasting and filtration, use of pollination drops to capture and transport wind-dispersed pollen, and the complex suites of mechanisms by which sessile organisms use mobile ones to transport their gametes (e.g., in animal pollination).
Sex6	Nonreproductive expressions and evolutionary derivatives of syngamy-facilitating actions	Inter/actions of sex5 expressed in contexts or ways that do not enable syngamy, and traits that evolved from them. In animals, includes nonreproductive sexual behavior, in both same-sex and different-sex contexts. In plants, examples include derived defensive use of ancestral pollinator rewards and multifunctional traits, such as wasp and bee-mimic orchid flowers that are pollinated by pseudocopulation (sex5 for plant/sex6 for insect) and may also, in a form of Batesian mimicry, repel herbivores (sex6 for plant).

Drawing across the range of sex concepts that exist in biology, we discuss pathways for biologists to broaden our perspectives, gain insights from concepts often considered specific to certain taxonomic groups, and use this to help us think about sex in ways that engage the breadth of biological diversity, rather than being limited by vertebrate-centric and other assumptions. To do this, we first provide some examples of how mammalian and vertebrate-centric assumptions infiltrate biological concepts and limit how and what we think about in biology, including the questions we ask, what we look for, and the ways we interpret data. Then, we provide two examples of concepts and terminology developed for “specialized” contexts in plants and/or modular metazoans and describe how these have the potential to be used more broadly. Lastly, we make suggestions for a path forward, building on the groundwork of the 2026 SICB Symposium “Sex Across Origins: Questioning Animal-centric Assumptions and Developing Integrative Frameworks” and the symposium manuscripts in this issue of Integrative and Comparative Biology.

## Identifying assumptions

We, as humans, bring a human and vertebrate bias with us as we interpret the world. Numerous assumptions that are rooted in our experiences as mammals and vertebrates limit our conceptualization of and communication about sexual processes, across biological scales and a range of sex concepts. Our cultural perspectives and the tools we have constructed to observe the world also inform these assumptions; the assumptions underlying multiple aspects of science vary across human cultural groups (Harding 1986, 1992; Kimmerer 2003, 2013; Kropotkin 2006; Winawer et al. 2007; TallBear 2013; O’Connor 2019; Prescod-Weinstein 2020).

Assumptions that underpin natural science practices can be described by the social science theoretical framework of master narrative theory (McLean and Syed 2015; Syed and McLean 2021). Master narrative theory explains how there are socially dominant (i.e., master) narratives that are socioculturally constructed explanations of how the world works (Syed and McLean 2021). While these narratives can be useful, they generally do not capture all of the variation and complexity that exists. Alternative narratives describe social norms or explanations of how the world works that differ from the master narrative (McLean and Syed 2015; Syed and McLean 2021). While master narrative theory was developed to explain social experiences in humans, here we use it as a lens to understand how our assumptions, oversimplifications, and overgeneralizations limit how we think in the western modern science practice of biology.

There are numerous ways that our mammalian- and vertebrate-centric biases influence how we, as humans, develop concepts in biology, including what we commonly do or do not explicitly name. Examples relevant for sex-related concepts include assumptions around the organization and interrelationship of reproduction with Sex0 and Sex1 and situating unitary body plans as normative, which influences how we conceptualize the organization of sex3 and sex4.

## Diplontic life cycles—often unnamed and situated as universal

In sexually reproducing eukaryotes, life cycles explain the organization of sex1, meiosis, and syngamy. Multicellular eukaryotes have three common sexual life cycle types: diplontic, haplontic, and haplodiplontic. Each describes the process of moving between syngamy and meiosis, with different amounts of time between these processes and variations in when mitosis occurs (Beukeboom and Perrin 2014). In diplontic life cycles, the diploid life stage is dominant, with gametes as the only haploid part of the life cycle. In haplontic life cycles, the haploid life stage is dominant, with the zygote as the only diploid part of the life cycle. And, haplodiplontic life cycles have components that are more complex than just a zygote or gamete for both the haploid and diploid stages; in multicellular organisms both the haploid and diploid stages are multicellular (Fusco and Minelli 2019).

Diplontic life cycles are often unnamed and universalized, with only “other” life cycles, including common ones such as haplontic and haplodiplontic, named as exceptions to this “rule.” This framing, or master narrative, situates diplontic life cycles as “normal,” and any other life cycle as “unusual.” Doing so not only limits how we think about life cycle functions, it also limits how we think about evolutionary processes. For example, organisms with multicellular haploid life stages (e.g., haplontic and haplodiplontic) can experience different selective pressures than those with only a multicellular diploid life stage. This is because multicellular haploid life stages require the expression of many genes, leading to selection against deleterious mutations and creating a greater potential for the selection of beneficial alleles (Otto and Gerstein 2008). The wide breadth of variation in life cycles, which goes beyond diplontic, haplontic, and haplodiplontic to include such variations as haplodiphasic, metagenic, and heterogonic (Fusco and Minelli 2019), further adds to the variation in how organisms experience selective pressures (Cronk 2022).

Illustrating how diplontic life cycles are usually centered in biology, it is common to teach students that meiosis makes gametes, situating gametes as the one type of haploid cell in a life cycle. However, this focus on meiosis making gametes (instead of simply creating new cells with half the ploidy of the parent cell, or discussing the other functions meiosis performs) reinforces the diplontic life cycle as normative. Another problem with centering diplontic life cycles as normative is that it situates gametes as the only type of reproductive cell, ignoring the existence of spores. Yet, spores are important reproductive cells in multiple types of life cycles, including haplodiplontic—e.g. all plants—and haplontic life cycles. For example, in ferns and bryophytes spores provide an important pathway for long-distance dispersal, unlike the more limited dispersal of their gametes (Haufler et al. 2016; Maciel-Silva and Porto 2014). In angiosperms and gymnosperms, the spores (macrospores or microspores) also play a vital role, growing into the multicellular haploid life stage that produces gametes (Fusco and Minelli 2019).

## Independence of sex and reproduction

The aforementioned life cycles all situate reproduction and sex1 as interrelated processes; this linkage is another master narrative about sex-related topics. However, sex does not inherently involve reproduction and reproduction does not inherently involve sex. The conceptual linking of these two concepts makes it difficult to discuss situations where they are not linked. And, it influences our ability to consider the developmental, evolutionary, and ecological limitations of assuming their linkage.

Sex0 is nonreproductive and sex1 can happen without reproduction. Horizontal gene transfer (HGT; sex0) involves nonmeiotic genetic recombination (Van Etten and Johnson 2026b) and happens broadly across the diversity of life (Ma et al. 2022; Van Etten and Johnson 2026a). HGT creates new genetic combinations, but not new cells or individuals. HGT is frequently discussed in regards to prokaryotes, protists, and other unicellular organisms (Van Etten and Bhattacharya 2020; Van Etten and Johnson 2026a). However, HGT also occurs in multicellular organisms, including between multicellular eukaryotes, such as through genes moving from animals to plants, or between different multicellular plants (Soucy et al. 2015; Van Etten and Bhattacharya 2020; Hibdige et al. 2021; Ma et al. 2022). This movement of genes can be particularly important for functions and phenotypes that can facilitate environmental adaptation, such as the evolution of land plants (Ma et al. 2022).

For sex1, ciliate conjugation is an example where meiosis and syngamy happen without reproduction, as the number of individuals does not change, yet afterwards both individuals involved are genetically different from the originals (Jiang et al. 2019). Conjugation in ciliates is different from bacterial conjugation because both cells change; in bacteria only one cell, the recipient, changes (Jiang et al. 2019).

Reproduction also commonly happens without sex. For instance, it happens through vegetative reproduction, as in plants, and fissiparous reproduction, as in some echinoderms and planaria (Maciel-Silva and Porto 2014; Fusco and Minelli 2019; Leria et al. 2019; Thuy et al. 2024). These processes involve reproduction without sex0, sex1, or any of the other processes involved in the sex concepts discussed by Warkentin et al. (2026).

## Fuzzy boundaries and changing conceptions of “sexual reproduction”

Along with clear examples of separate and interrelated sex and reproduction, there are also fuzzy boundaries between asexual and sexual reproduction. The process of parthenogenesis, in which a gamete grows into a new individual without fusing with another gamete, exists in this fuzzy boundary space (Fusco and Minelli 2019). There are numerous forms of parthenogenesis that occur across taxa and involve different cytogenetic mechanisms, including in plants and animals (Koltunow 1993; Richards 2003; Naumova 2008; Cornaro et al. 2023; Blanc et al. 2025). Biological literature often differentiates between “mitotic parthenogenesis” and “meiotic parthenogenesis” (Blanc et al. 2025). “Meiotic parthenogenesis” involves processes clearly derived from meiosis (Cornaro et al. 2023). “Mitotic parthenogenesis" is usually considered to use only mitotic processes (Cornaro et al. 2023). However, the mechanisms involved in “mitotic parthenogenesis” vary, and Blanc et al. (2025) argue that most, if not all, are likely meiotically derived in metazoans. In plants, there are many ways to produce seeds without typical meiosis and syngamy (Richards 2003; Cornaro et al. 2023); these are often all labeled as parthenogenesis, or with the plant-specific term of apomixis (asexual reproduction via seeds). Some of these are clearly meiotic, as when endoreplication restores diploidy before or after meiosis (Cornaro et al. 2023). Others appear to involve a modified version of meiosis, aborting one of the cell divisions as occurs in many metazoans (Blanc et al. 2025); see Coranaro et al. (2023) and Kultonow (1993) for pathway descriptions. However, the parthenogenesis pathway of apospory produces embryos that grow from gametes that are cytologically produced through mitosis. Nonetheless, the development of these gametes, and the embryos they produce, relies on the developmental environment created for meiotically produced cells, which fail to form or develop (Koltunow 1993; Cornaro et al. 2023). Another form of asexual reproduction by seed, adventitious embryony, only uses mitosis (Wilms et al. 1983; Xu et al. 2021); however, the embryo does not develop from a gamete, and thus is not parthenogenetically formed. Additionally, the embryo is nutritionally dependent on endosperm created by the fertilization of the central cell in most species (Wilms et al. 1983; Asker and Jerling 1992; Koltunow 1993; Xu et al. 2021). Therefore, unlike vegetative reproduction, asexual adventitious embryony is usually not fully independent of sex1.

There is no consensus on which of these uniparental reproductive processes should be considered asexual or sexual. Some classify meiotic parthenogenesis as asexual reproduction (Mirzaghaderi and Hörandl 2016; Blanc et al. 2025); others argue that meiotic parthenogenesis is a sexual process, as it is derived from meiosis, i.e. a part of sex1 (Fusco and Minelli 2019). The latter argument is supported by the occurrence of genetic recombination in some meiotic parthenogenic pathways, yielding offspring genetically similar to those produced through self-fertilization (Fusco and Minelli 2019; Warkentin et al. 2026). Furthermore, some types of reproduction, such as polyembryony (when a single embryo divides to create multiple genetically identical siblings) could either be classified as sexual, with parents producing a clonal set of offspring that are genetically different from themselves, or as a multi-stage reproductive cycle with an embryo that reproduces asexually (Fusco and Minelli 2019).

In addition to these fuzzy boundaries between sexual and asexual reproduction, the haplodiplontic life cycle demonstrates how the way we conceptualize life cycles in biology has changed over time. Previously, scientists considered this life cycle to include both asexual and sexual components (de Bary and Strasburger 1877; Sandford 2023), and student conceptions can follow this earlier biological understanding (Casper and Dockendorf 2023). However, biologists have now clarified that the haplodiplontic life cycle (and the related haplodiphasic life cycle) is entirely sexual: the spores are created through meiosis, which later leads to the genetic diversity in gametes that are made by mitosis (Fusco and Minelli 2019). Therefore, the meiotically produced spores are an important part of the sexual life cycle. In contrast, for life cycles that include both asexual and sexual reproduction, there is no change in ploidy between the sexually produced adult and its asexually produced offspring (Fusco and Minelli 2019).

## The usually unnamed unitary body plan: Situating sex3 in bodies

Body plans describe the structural organization and levels of independence of biological units and subunits. Another common but often unstated perspective in biology is the master narrative that it is easy to define an individual. This is only true when genetic, developmental, structural, and functional units align to form a single coherent individual body that lives, reproduces, and dies as an integrated whole, as in humans and many other animals (Vuorisalo and Tuomi 1986). Organisms that are clonal, colonial, or have other more complex body plans are situated as unusual while unitary body plans (such as our own) often go unnamed. The strength of the assumption of unitary body plan normativity is illustrated by the late definition of modularity as a concept in biology. While the published record of discussions regarding the challenges of defining an “individual” in plants dates back at least to the 1700s (White 1979), in the latter half of the twentieth century biologists began to unify language for modularity in plants, including using the term “modular” (Shinozaki et al. 1964; Harper and White 1974; White 1979; Harper 1980; Barthélémy and Caraglio 2007). Modularity was then adapted for broader use, particularly in modular metazoans such as corals (Hughes and Jackson 1985; Vuorisalo and Tuomi 1986; Wasson and Newberry 1997; Hiebert et al. 2021).

Situating unitary body organization as normal makes it challenging to think about sex3 in modular organisms, such as plants or modular metazoans. Modular organisms have repeating units across one or more levels, which can differ in their sex expression. Therefore, hermaphroditism can occur in many different ways. Identifying the sex3 and sex4 of a modular organism requires specifying the focal level/s of organization and parsing out what is considered an individual. Both botanists and biologists who work with modular metazoans have grappled with ways to represent organization across levels of modularity, but there is no unified framework (Vuorisalo and Tuomi 1986; Cox 1988; Wasson and Newberry 1997; Hiebert et al. 2021; Pannell 2023). We consider the challenges of integration and possibilities for broader use of such representations below (see Organization of gamete production in bodies: Unitary and modular organisms).

## The utility of naming master narratives

The topics discussed here name only a few ways our mammalian and vertebrate assumptions influence sex-related topics in biology and how biology is used more broadly in society. Considering the implicit and commonly held assumptions that underpin biological language and conceptions is necessary to understand organisms across diverse taxa and can help us to understand ourselves and more closely related organisms better as well. These human-centric assumptions are also embedded directly in the language we use (Sandford 2023; Subramaniam and Bartlett 2023; Subramaniam 2024). For example, several of Linnaeus’ terms in *Systema Naturae*, which continue to be used in botany, reference marital arrangements (Schiebinger 2013; Sandford 2023). A small sampling of these include how husbands and wives rejoice in the same bed chamber in monoclinia, have separate bed chambers but share the same house in monoecia, and live in separate houses in dioecia (Sandford 2023). Understanding the history embedded in the language of biology helps us understand the social context that has formed our current knowledge, potentially aiding us in identifying and addressing limitations created by these perspectives.

Considering the history and roots of potentially problematic terms in biology, some have called for replacing them. For instance, arguments have been made to replace the word *hermaphrodite* (Driessen et al. 2024) due to its history of use as a slur for intersex and queer people (Costello 2016; Viloria and Nieto 2020; Brooks 2026). However, others discuss the reclamation of this word by some people with variations of sexual development, thus perspectives on the term vary (Costello 2016; Viloria and Nieto 2020). While changing language systemically throughout biology is important in some cases, it is a complex process that requires cooperation across the field and a clearly defined new word that is not already in use. Moreover, knowing the older language is still necessary to understand work published prior to the language change (see Casper 2026 for a longer discussion of this topic). In many cases, discussing the history of biological language may be more feasible and also, usefully, require us to grapple with rather than avoid the historical roots of current biological knowledge.

Although we may always rely on simplifications or generalizations in certain aspects of biology, naming and understanding them explicitly situates our assumptions and contextualizes what might otherwise be understood as “normal” or universal. Knowing and using the biological terms for our own life cycle, body plan, and other biological features is vital for understanding that there are multiple ways of being, rather than framing variation as “normal” versus “other.” While we may need to invent new terms or clarify ambiguous terms in some cases, the language we need may also exist in biology but simply be unfamiliar. For one example, *hermaphroditic* is a commonly used word, even in introductory biology, but its parallel, *gonochoric*, for an organism that only makes one type of gametes, is not (see Casper 2026 for a longer discussion of this topic). For another, one of the authors only learned the term *amphigonic* while preparing the talk on which this paper is based, yet it names the common and familiar form of sexual reproduction in which gametes from two different parents combine. Through naming these normative—but not actually universal—processes we can begin to reveal the assumptions and overgeneralizations implicit in our discussions and conceptualizations of biology, and from there start to understand how these assumptions limit us.

## Applying “specialized” frameworks more broadly

Identifying the problems caused by mammalian and vertebrate-centric assumptions and overgeneralizations is just a first step towards addressing them. We need shifts in scientific thought and language to navigate these challenges. While some require new language or concepts, some existing “specialized” concepts could be utilized more broadly and/or offer starting points to develop more biologically inclusive frameworks. Here we discuss two: sexual modes in modular organisms and quantitative gender.

## Organization of gamete production in bodies: Unitary and modular organisms

**Fig. 1:**
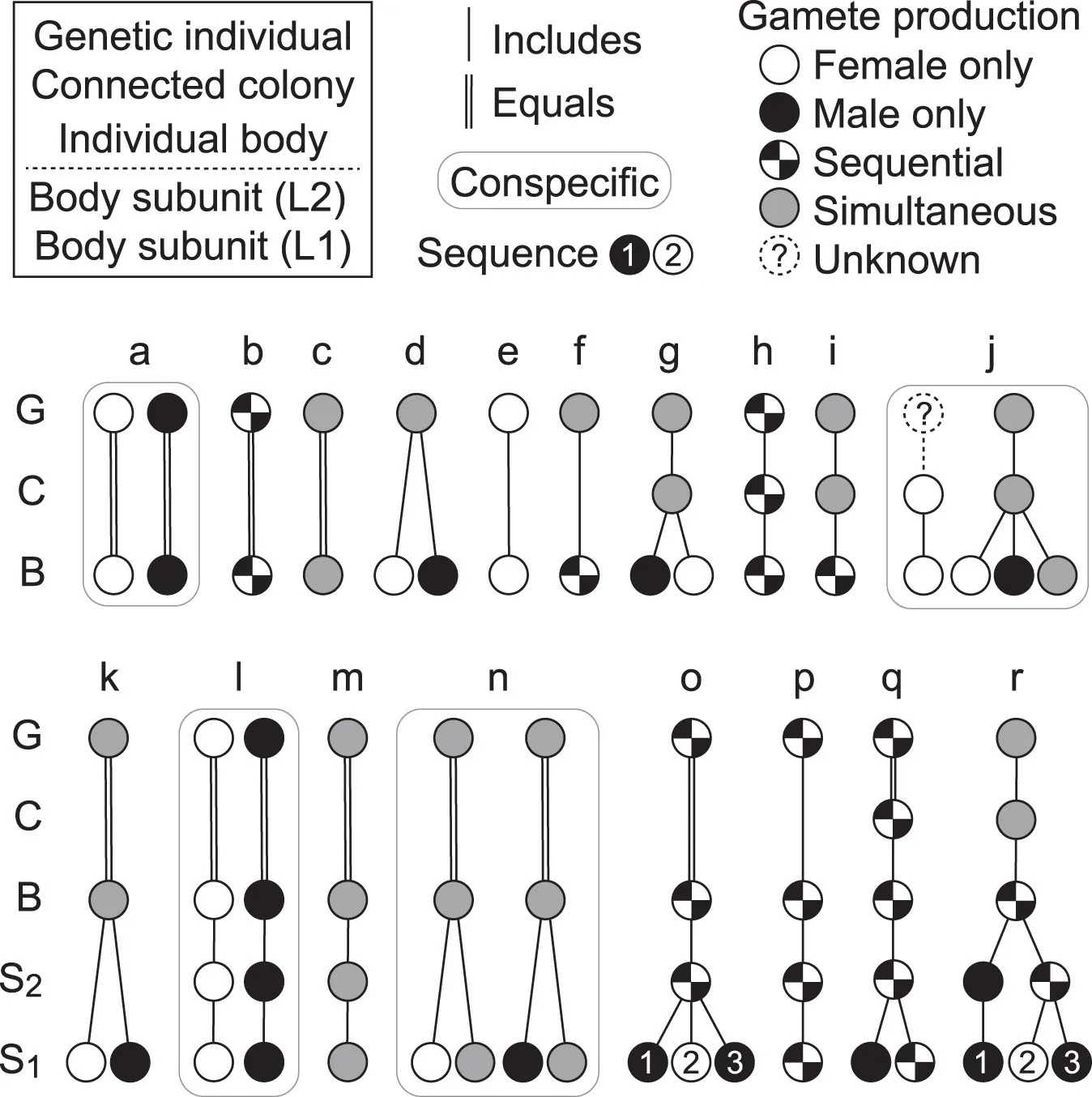
A graphical system for mapping anisogamete-production strategy (sex3) across levels of organization in unitary and modular organisms, with examples. The system is illustrated with 3 levels (a–j) and 5 levels (k–r), but could be expanded or condensed to show more or fewer levels of modularity. Starting from actually or potentially independent individual bodies (B, ramets, zooids, etc.), higher levels can include colonies (C, physically connected groups of clonal bodies) and genetic individuals (G, genets, which may comprise one or more colonies, as in corals, or separate individual clonemates, as in aphids). Bodies can include multiple gamete-producing subunits (S_1_, subunit level 1), such as flowers, which may be grouped into higher-level structures (S_2_, subunit level 2), such as inflorescences. Circle fill represents sex3 at each modular level: white for female, black for male, white and black for sequentially hermaphroditic, grey for simultaneously hermaphroditic (cosexual). Dashed circle with ? indicates that sex3 is unknown, as is common for the genets of colonial organisms. Single lines connect modular units across nested, hierarchical levels of inclusion. Double lines represent identity across levels; if all reproduction is sexual, genetic individuals have only one body and G = B. Grey rectangles enclose multiple conspecific sex3 alternatives, where those exist. If sequential hermaphroditism at one level is generated by sequential production or function of female and male units at a lower level, the sequence can be numbered. A sampling of sexual systems is illustrated as follows: (a) human, a unitary gonochorist; (b) clownfish *Amphiprion percula*, a unitary protandric hermaphrodite; (c) black hamlet fish *Hypoplectrus nigricans*, a unitary cosexual; (d) water flea *Daphnia magna*, gonochoric individuals in cosexual clones (Cornetti and Ebert 2021); (e) whiptail lizard *Aspidoscelis uniparens*, all-female parthenogenetic clonal lineage (Lutes et al. 2010); (f) starfish *Nepanthia belcheri*, fissiparous with protandric development (Wasson and Newberry 1997); (g) pleurobranch *Rhabdopleura compacta*, a colonial hemichordate with gonochoric zooids (Wasson and Newberry 1997); (h) ascidian tunicate *Botyllus schlosseri*, protogynous zooids sexually synchronized within and across clonal colonies in the same environment (Wasson and Newberry 1997); (i) bryozoan *Bowerbankia gracilis*, protandric zooids unsynchronized within colonies; (j) coral *Porites astreoides*, female colonies and cosexual (polygamomonoecious) colonies containing female, male and cosexual polyps (Guest et al. 2012); (k) limber pine *Pinus flexilis*, nonclonal (i.e., all reproduction is sexual) monoecious tree, with male and female cones; (l) *Ephedra viridis*, nonclonal dioecious shrub with inflorescences of male or female flowers (Van Drunen and Dorken 2014); (m) foxglove *Digitalis purpurea*, a herb with inflorescences of cosexual flowers; (n) swamp tupelo *Nyssa biflora*, a polygamodioecious tree that has either female and cosexual or male and cosexual flowers; (o) *Cupania guatemalensis*, a monoecious tree with sexually synchronized inflorescences of three flower morphotypes that mature in a male, female, male sequence (Bawa 1977); (p) avocado *Persea americana*, a heterodichogamous tree, horticulturally propagated by grafting, with inflorescences of sequentially hermaphroditic flowers that are temporally offset between two types (A, female in the morning then male in the afternoon and B, female in the afternoon then male in the morning) (Solares et al. 2023; Alder 2025), (q) bristly sarsaparilla *Aralia hispida*, a shrub with small genets of sexually synchronized colonial ramets that have inflorescences of male and phase-linked protandric flowers (Barrett 1984); (r) *Sagittaria trifolia*, a colonial monoecious herb that can have an initial male inflorescence followed by a series of protogynous inflorescences with female and male flowers (Huang et al. 2002).

In unitary organisms, individuals can produce either one gamete type (gonochoric males and females) or both types (sequential and simultaneous hermaphrodites) (Leonard 2018; Fusco and Minelli 2019). However, the organization of gamete production in modular organisms is more complicated (Fig. 1). Genetic individuals (genets) can have multiple gamete-producing subunits, which may differ in the type/s of gametes they produce and the timing of their gamete production (Cox 1988; Wasson and Newberry 1997). Moreover, these subunits may be nested within genets at multiple levels of structural organization (Vuorisalo and Tuomi 1986). Higher-level subunits may be physically independent (e.g., individual aphids in a clonal lineage) or connected (e.g., polyps in a coral, aspen tree ramets in a grove), but are able to survive if disconnected. Lower-level units are semi-independent parts of a larger body (e.g., flowers); these subunits can differ in their gamete production but cannot survive as independent organisms. This nested hierarchy of (semi)independent gamete-producing subunits creates many different ways to organize gamete production spatially and temporally, as well as multiple ways to achieve similar higher-level sex3 conditions, and the specifics of sexual organization have important implications for development, evolution, and ecology.

Considering the organization of sex3 at multiple levels is crucial for modular organisms. However, any framework to describe sex3 across multiple levels could also encompass organisms with just one level, facilitating comparative work. Although it may seem unnecessary to explicitly name the single level of sex3 organization in unitary organisms, doing so emphasizes that their body plans are not universal, and situates unitary organisms within the broader realm of organismal diversity. In contrast, leaving the unitary body plan unnamed positions it as the implied norm and modularity as an oddity or exception. Language and frameworks for discussing multi-level sex3 have been developed for plants (Cox 1988) and for modular metazoans (Wasson and Newberry 1997), but there is as yet no general comparative framework. We introduce the metazoan framework and discuss some challenges in developing a broader framework below.

Wasson and Newbury (1997) developed a multilevel framework for discussing sex3 in unitary and modular metazoans, which lays a foundation for considering sexual organization across levels of modularity. They consider three levels of organization: individual bodies (which they call “modules”), colonies of physically connected individuals, and genetic individuals (genets). They label sex3 at each level with a letter: H for simultaneously hermaphroditic, S for sequentially hermaphroditic, and G for gonochoric, in a Genet-(colony)-Module sequence. Thus, a unitary organism can be described with one letter (e.g., G), a non-colony-forming clonal organism with two letters (e.g., HG), and a colonial clonal organism with three letters (e.g., HhG). The lower-case letter for colony-level sex3 indicates colonies are a level only sometimes present between individual and genet.

While this framing is conceptually applicable beyond metazoans, such as for plants, algae, and fungi, its practical implementation is more complicated (Cox 1988; Wasson and Newberry 1997). Even considering just modular metazoans and plants, challenges to developing a broadly applicable conceptual framework include variation in the organization of sex3 and its modularity across different life cycles, variation in the number of levels of modularity, and the complexity of identifying parallels in levels of organization. The examples below focus on plants and modular metazoans for brevity, but a general comparative framework should also be applicable to describe sex3 across the forms of modularity in other organisms, such as fungi and algae.

Life cycles influence the organization of sex3 across levels of modularity. For instance, plants have haplodiplontic life cycles with alternating haploid (gametophyte) and diploid (sporophyte) generations. In homosporous plants, such as mosses, sex3 is determined by, as well as expressed in, the gametophyte generation; sporophytes play no role in sex3 organization and are considered sexually indeterminate. However, in heterosporous plants, such as angiosperms and gymnosperms, sex3 is determined by the sporophyte generation but expressed in the gametophyte generation. Thus, considerations of sex3 organization in these plants integrate both sporophyte and gametophyte generations. While animals are diplontic, and many have unigenerational life cycles, others have more complex, multigenerational life cycles of various sorts. For instance, metazoans can have life cycles alternating generations of equal ploidy with obligately asexual and sexual reproduction (i.e., metagenic cycles), or generations with parthenogenetic and amphigonic reproduction (i.e., heterogonic cycles), or gonochoric and hermaphroditic generations (i.e., heterogenic cycles), or solitary and colonial generations (Fusco and Minelli 2019). Thus, considerations of potentially multilevel sex3 can also require integrating across multiple generations in animals.

As well as the need to integrate considerations of sex3 across multigenerational life cycles, another challenge is navigating systems with different—and sometimes high—levels of modularity. Wasson and Newbury (1997) acknowledge that their letter system for multilevel sexual modes would become increasing unwieldly at higher numbers of levels. For instance, angiosperm sex3 can vary at genet, colony, ramet, inflorescence, and flower levels (Vuorisalo and Tuomi 1986; Cox 1988).

Moreover, it may be challenging to identify which levels of modular organization are parallel across different types of organisms, although the genet concept is broadly comparable across life forms, including modular metazoans and plants. Wasson and Newbury (1997) situate angiosperm flowers as “modules” comparable to animal zooids, focusing on their similarity as the lowest level of sex3 expression, and then situate “the plant” as comparable to a metazoan colony. However, flowers are never independent of plants, and plants often reproduce vegetatively, generating colonies of ramets. Thus, to us, the ramet seems a better parallel to zooids. In this framing, inflorescences and flowers would be lower-level subunits with no direct parallel in the Wasson and Newbury (1997) framework. Moreover, their tripartite classification for each level (G, S, or H) does not include all the sexual possibilities of either animals or plants. For instance, it cannot describe organisms that include hermaphroditic and male or female units at any level of organization. Adding more letters to include such combinations could also be increasingly unwieldy. Thus, while useful in allowing comparisons across modular metazoans, illustrating the need for a broader comparative framework, and revealing some of the challenges of such a task, Wasson and Newbury’s (1997) approach may not be sufficiently scalable.

Considering variation in the number of levels of modularity and challenges in identifying parallels across organisms, another approach could be to build a scalable framework to describe the nested hierarchy of sex3 across any number of levels, allowing flexibility in identifying parallels across taxa according to the focus of an investigation. We developed the approach illustrated in Fig. 1 as a starting point for such a framework. However the challenges of broad comparisons are resolved, explicitly acknowledging multilevel sex3 organization recognizes important dimensions of sexual variation and provides opportunities to discuss differences and similarities among unitary and modular organisms.

An additional benefit of considering sex3 characteristics across organizational levels is that it helps to differentiate between terms that are sometimes erroneously used as synonyms, such as gonochoric and dioecious, or hermaphroditic and monoecious. Both gonochoric and hermaphroditic convey information about a single level of organization. Gonochoric individuals make only one type of gamete (Fusco and Minelli 2019), thus if within-individual subunits are present they must make the same gamete type. Hermaphroditic individuals produce both macrogametes and microgametes but may do so in many ways—sequentially or simultaneously, with unitary organization, or through many different patterns of modular sub-structure. In contrast to gonochoric and hermaphroditic, the botanical terms dioecious, monoecious, and synecious provide information about sexual characteristics at two levels: the individual (plant) and a lower-level subunit, e.g. flower in angiosperms or cone in gymnosperms (Jackson 1928). Thus, a dioecious species is necessarily modular, and gonochoric or unisexual at two levels. A monoecious species is hermaphroditic at the plant level, with gonochoric/unisexual subunits of both sexes. And a synecious plant is hermaphroditic across two levels (Nath and Asthana 2001; Beukeboom and Perrin 2014). Moreover, these terms apply specifically to species with sporophytic sex3 determination. The parallel terms dioicous, monoicous, and synoicous refer to sexual organization in species with gametophytic sex3 determination (Jackson 1928; Maciel-Silva and Porto 2014; Coelho et al. 2018; dos Santos et al. 2026).

These terms do not address sexual organization at higher levels (i.e., genet and potentially colony, in species with clonal propagation), across multiple lower levels (e.g., inflorescence structure), or in sex expression timing. However, there is rich array of additional botanical terminology that conveys greater specificity (Jackson 1928), some of which may be useful for other modular organisms. For instance, synchronous dichogamy refers to plants that are sequential hermaphrodites at multiple levels, i.e. B plus S1 and/or S2 from Fig. 1 (Lloyd and Webb 1986), fitting within the need Wasson and Newbury (1997) identify for combining information about temporal sex expression and modular structure. Botanical and modular metazoan researchers have both developed language and conceptual frameworks to describe multiple aspects of sexual variation. Developing an organismally inclusive comparative framework to discuss sexual organization across levels of modularity will require collaborative work across these specialties, and with specialists focused on other organismal groups.

## Quantitative gender

Quantitative gender is another concept that was developed for a specific group of organisms, i.e. plants, but that has broader utility. Many plants, particularly angiosperms, are hermaphroditic, either sequentially or simultaneously, and the proportion of each gamete type that a simultaneous hermaphroditic plant produces can change over time. To describe this variation, Lloyd (1980) developed the concept of quantitative gender, which is distinct from human-centric uses of gender (see McDonough-Goldstein et al. 2026 for a further discussion of “gender” in biology). Because of the other meanings of the term, some botanists argue against using “gender” for the botanical concept of quantitative gender, whereas others argue for its continued usage (Diggle 2023; Oberle and Fairchild 2023; Pannell 2023). Here, we focus on the utility of the concept of quantitative gender; see Casper (2026) for a discussion of varied perspectives regarding the continued use of the term “gender” to name the concept.

There are two types of quantitative gender: phenotypic gender and functional gender (Lloyd 1980). Both are numbers that range from 0 to 1 and can change over time. Phenotypic gender describes a plant’s allocation to eggs and sperm; a plant with a phenotypic gender of 0.7 produces 70% eggs and 30% sperm within the specified time frame (Lloyd 1980; Pannell 2023). Functional gender describes how the plant’s gametes contribute to the next generation, and is influenced by the population the plant lives in and mechanisms of gamete or gametophyte transfer (Lloyd 1980; Pannell 2023; Sandford 2023). Thus, for any given time frame, an individual has both functional and phenotypic genders, which can differ. And, each type of gender will range from 0 to 1 for a hermaphroditic individual. For comparative work, gonochorists can also easily be characterized, having identical phenotypic and functional genders that are 0 for individuals that produce sperm and 1 for individuals that produce eggs. Thus, both types of quantitative gender can be applied to any anisogamous organism, with a particular utility for hermaphrodites.

Using this system, an individual that does not produce any gametes would have no gender. Depending on which concept of sex is considered—i.e., traits associated with gamete type/s produced (sex4) or actual gamete type/s produced (sex3, Table 1)—the individual may or may not have a sex. This possibility for a null gender, and potentially sex, may seem cognitively jarring at first, but simply reveals a socially ingrained idea that sex and gender are ever-present characteristics of an organism.

For the many plants that do not fit neatly into boxes of “male,” “female,” or “hermaphrodite,” such as *Plocama pendula*, a woody shrub endemic to the Canary Islands, the concept of quantitative gender can be useful even if one does not assign numbers. Anderson et al. (2021) characterized *P. pendula* individuals using three categories: females and two morphotypes of hermaphrodites that differ in their morphology and functional gender, although all flowers appear morphologically hermaphroditic (i.e., their sex4 is hermaphroditic). Anderson et al. (2021) then studied the fruit production of each category. In this way, they provide an interesting application of the concepts of phenotypic and functional gender without needing to delve into the difficult data collection or math required to formally calculate these characteristics. *P. pendula* also exemplifies a species where the sex4 of an individual does not predict its sex3.

In addition to usefully characterizing both gamete production and offspring, one could also apply quantitative gender across different levels of modularity, an idea also suggested by Cox (1988) and Pannell (2023) that has yet to gain traction. This could be particularly useful for plants that have multiple types of reproductive structures on the same plant, such as angiosperms with some mixture of male, female, and hermaphroditic flowers, as one could assign gender numbers to each level (e.g., adding the numbers to diagrams such as those in Fig. 1). For example, a monecious plant could have a gender that varies from 0–1, with inflorescences that change from 0 to 1 as different flowers open, composed of flowers that are either 0 or 1. A dioecious plant would either be 0 or 1 across all levels of modularity. A synecious plant would have values between 0 and 1, for each level of modularity, with the number for each level of modularity capturing the variation in the proportional investment in different types of gametes. One could also use the concept of quantitative gender across levels of modularity to conceptually consider the evolutionary and developmental ramifications of different investment strategies, as well as map the differential influence of environmental sex determination factors.

## Synthesis

In considering interdisciplinary work, Harraway, Barad, and others discuss using one discipline as a prism to help identify and break apart assumptions in another discipline, to identify better paths forward (Barad 2006; Haraway 2018; Appedu 2025). This same approach can be used to help break apart common oversimplifications and overgeneralizations in biology, by putting concepts through the “prism” of a wide range of organisms. This practice can help us ask: how broadly does a concept work, what can we learn from organisms where it fails, and what are the possibilities for creating concepts that work more broadly? For example, lions are often used in biology textbooks as an example of sexual dimorphism. If we use the idea of a binary sexual dimorphism that aligns gamete type produced with traits associated with gamete production and organismal actions that bring gametes together for sexual reproduction, or sexes 3–5 (Table 1), we can use some overgeneralizations to fit lions into this idea—although there are multiple ways they don’t actually fit (Chavan 1981; Gilfillan et al. 2017; Anderson et al. 2024). But, when we bring in other organisms, such as a wide range of invertebrates, plants, fungi, and protists that simply cannot be forced into a box that expects sexes 3–5 to be aligned into neat binaries, it can help us start to think about the broader possibilities that exist.

Collectively, the other papers in this volume, along with the examples we have discussed, demonstrate the vital need for conversations and conceptualizations that bridge boundaries across taxa, scales of thinking, conceptual focal areas, and more. Humans enter the world with biases based on our own biology, which makes it more difficult for us to understand organisms that are different from us, particularly those—such as plants, protists, algae, fungi, and modular metazoans—that are very different from us. The oversimplifications and overgeneralizations that we are primed for through our lived experience are often reinforced through introductory and upper-level biology courses, and even siloed research focused on a specific type of organism (Bazzul and Sykes 2011; Fuselier et al. 2018; Ray King et al. 2021; Casper et al. 2022, 2025; Zemenick et al. 2022, 2023; Spaulding and Fuselier 2023). However, considering how common assumptions and concepts do or do not work across many types of organisms helps to reveal our biases and identify points that need more clarity, including potentially irreconcilable conflicts that need naming, as well as tractable places for growth and integration. Through this process we can ask: what breaks apart, what is useful, and what is newly visible through a broader organismal lens. These considerations are vital for both researchers and instructors, because how we teach biology sets up foundational societal knowledge and beliefs that underpin how we approach understanding the world (Rende Mendoza and Johnson 2024; Adams et al. 2026; Blake et al. 2026; Eddy et al. 2026a, 2026b; Sedlacek et al. 2026). The discussions in this paper, as well as the others in this issue serve only as a starting point for these conversations.

## Data Availability

Data sharing is not applicable to this article, as no new data were created or analyzed in this study.

## Notes

From the symposium “Sex across origins: Questioning animal-centric assumptions and developing integrative frameworks” presented at the annual meeting of the Society for Integrative and Comparative Biology, January 3-7th , 2026.
